# The effect of temperature increase on the development of *Rhodnius prolixus* and the course of *Trypanosoma cruzi* metacyclogenesis

**DOI:** 10.1371/journal.pntd.0006735

**Published:** 2018-08-15

**Authors:** Laura D. Tamayo, Felipe Guhl, Gustavo A. Vallejo, Juan David Ramírez

**Affiliations:** 1 Centro de Investigaciones en Microbiología y Parasitología Tropical (CIMPAT), Facultad de Ciencias, Universidad de Los Andes, Bogotá, Colombia; 2 Laboratorio de Investigación en Parasitología Tropical, Facultad de Ciencias, Universidad del Tolima, Ibagué, Colombia; 3 Grupo de Investigaciones Microbiológicas-UR (GIMUR), Programa de Biología, Facultad de Ciencias Naturales y Matemáticas, Universidad del Rosario, Bogotá, Colombia; Universidade Federal de Minas Gerais, BRAZIL

## Abstract

The increase in the global land temperature, expected under predictions of climate change, can directly affect the transmission of some infectious diseases, including Chagas disease, an anthropozoonosis caused by *Trypanosoma cruzi* and transmitted by arthropod vectors of the subfamily Triatominae. This work seeks to study the effects of temperature on the development of the life cycle, fertility and fecundity of the insect vector *Rhodnius prolixus* and on the metacyclogenesis of *T*. *cruzi*. All of the variables were subjected to 3 temperatures: 26°C, 28°C and 30°C. Hatching time was evaluated, along with time to fifth instar, time to adult, fecundity studied using the e-value, and egg viability during the first 3 reproductive cycles. In addition, the amounts of metacyclic trypomastigotes of the TcI and TcII DTUs in *R*. *prolixus* were evaluated from days 2 to 20 at two-day intervals and from weeks 6 to 8 post-infection. Decreases were observed in time to hatching (15–10 days on average) and in time to fifth instar (70–60 days on average) and transition to adult (100–85 days on average). No significant differences in egg viability were observed in any of the reproductive cycles evaluated, but an increase in fecundity was observed at 30°C during the third reproductive cycle. At 30°C, there was also an increase in the number of infective forms and a decrease in the time at which metacyclic trypomastigotes were detected in the rectal ampulla of the insects for both TcI and TcII. According to these results, the expected temperature increase under climate change would cause an increase in the number of insects and a greater probability of infection of the parasite, which affects the transmission of Chagas disease.

## Author summary

Chagas disease is an anthropozoonosis caused by the flagellated protozoan *Trypanosoma cruzi* and mainly transmitted through the infected faeces of insects of the subfamily Triatominae. Because these insects are sensitive to climatic conditions, it is expected that disease transmission may be affected by the increase in global land temperature, predicted under climate change. Therefore, we wanted to evaluate the effect of temperature increase on the development, viability of eggs and fertility of *R*. *prolixus*, the most important vector insect in Colombia, and on the development of the parasite within this insect. We observed a decrease in the development time of *R*. *prolixus* and an increase in the number of infectious forms of *T*. *cruzi* in the insect as the temperature increased. These results suggest that if the temperature increases as expected, there may be an increase in the number of insects that can transmit the disease, as well as an increase in the likelihood of infection due to the increase in the number of infectious forms. Our data contributes to the understanding of the possible effects of the expected temperature increase under climate change on Chagas disease transmission and can be used to make predictive models that can more accurately predict the future of Chagas disease.

## Introduction

According to climate prediction models, it is expected that by the end of the 21st century, the global terrestrial temperature will increase between 0.3°C and 4.8°C with respect to the average temperature observed between 1986 and 2005 [1]. This increase can affect the transmission of infectious disease agents transmitted by vectors because both insects and vertebrate reservoirs are sensitive to climatic conditions [2]. The alteration in transmission occurs because the temperature and rainfall expected under the effect of climate change can affect the range, proliferation, viability and maturation rates of vectors, pathogens and reservoirs. [3]. Several studies have reported variations in the transmission of diseases such as malaria [4], dengue [5,6] and leishmaniasis [7,8]. In the case of malaria, it has been observed that the time taken by *Plasmodium*, within the vector mosquito, to pass from gametocytes to infective sporozoites decreases when the temperature of the environment increases [9]. Likewise, a decrease in the development time of the insect vector has been reported, which leads to an increase in the number of insects per season and consequently to a higher transmission rate [10]. In some countries, such as Colombia, Venezuela, Guyana and Peru, there has been a resurgence or intensification of endemic and epidemic malaria that correlates with the phenomenon of the El Niño Southern Oscillation (ENSO) [11, 12]. Likewise, changes in the distribution of leishmaniasis and Dengue virus vectors that may lead to an increase in the risk of transmission of these diseases have been predicted [5, 7].

Another parasitic disease transmitted by vectors that could be affected by climate change is Chagas disease, an anthropozoonosis caused by the flagellated protozoan *Trypanosoma cruzi*, which is transmitted to humans and other mammals mainly through the infected faeces of insects of the subfamily Triatominae [13]. The disease is a serious public health problem; it is estimated that more than eight million people are infected in Latin America [14]. Despite efforts to control vectors in various regions of Latin America, the World Health Organization estimates that 5,274 new cases occur annually due to vector transmission in Colombia, 933 in Honduras and 873 in Venezuela, countries in which *Rhodnius prolixus* is the main transmitting vector of the disease [15].

More than 150 species of triatomines have been reported in Latin America, of which 10 are considered primary vectors of the parasite because they colonize houses, while another 20 are considered secondary vectors because they invade human habitations from their peri-domestic or wild habitat. [16] *R*. *prolixus* is present in both domestic and wild transmission cycles, which is why it is imperative to design very specific vector control strategies [17]. The life cycle of this vector includes 5 nymphal stages and an adult stage, all obligate haematophagous. None of the nymphs can develop into another stage without having fed at least once. In addition, adults cannot produce eggs without ingesting blood, as it is essential for this process [18]. *R*. *prolixus* is considered the most important vector of *T*. *cruzi* transmission in Colombia, Venezuela and most Central American countries [13,19]. Also, different outbreaks of oral transmission have been reported in Colombia and Venezuela incriminating *R*. *prolixus* [20, 21, 22].

The effect of temperature over *R*. *prolixus* life cycle has been barely studied. It is well known that under laboratory conditions, the temperature range for eclosion and molting of *R*. *prolixus* was reported to be between 16–34°C [23].No development was observed at 15°C and 35°C [24, 25]. Development is generally studied at constant temperatures between 25 and 28°C and about 70% humidity or unspecified conditions of ambient temperature and humidity. However, temperature and humidity in insect habitats may differ considerably and vary according to circadian and seasonal patterns. R. prolixus is found mainly in Colombia and Venezuela from 0 to 2,600 m above sea-level, in regions with annual median temperatures from 11 to 29°C and 250 to 2,000 mm annual precipitation [26, 27, 28].

The biological cycle of *T*. *cruzi* in the vertebrate host begins with contact of the faeces of a triatomine infected with metacyclic trypomastigotes. The parasite penetrates the cells by forming a vacuole bound to lysosomes, then escapes from the vacuole and differentiates into amastigotes, which reproduce in the cytoplasm by binary fission. Subsequently, amastigotes differentiate into metacyclic trypomastigotes that are mobile. These latter forms of the parasite lyse the host cell, disperse and can infect other cells. When insects ingest blood from an infected mammal, trypomastigotes in the blood differentiate into epimastigotes, and some develop into spheromastigotes. The epimastigotes divide in the midgut by binary fission and migrate to rectal ampulla, where they are transformed into metacyclic trypomastigotes (a process called metacyclogenesis), which are eliminated in the faeces of the triatomine [29]. *T*. *cruzi*, in turn, has high genetic variability, which has been subdivided by international consensus into six Discrete Typing Units (DTUs), TcI—TcVI, including a DTU associated with anthropogenic bats and called TcBat [30, 31, 32]. In the northern countries of the southern cone, TcI and TcII are the most frequent DTUs in humans, vectors and reservoirs [30]. In Colombia, it is observed that TcI and TcII are the most frequent DTUs in *Rhodnius prolixus* [17].

Efforts to predict the future of transmission of Chagas disease under the effects of climate change have been scarce and have focused, above all, on trying to establish possible changes in the distribution of vectors through mathematical models [33, 34, 35, 36, 37, 38]. In general, the results obtained have been varied and seem to depend on the vector species, which is why more studies are needed on the physiology of the parasite and the vector to enable more reliable predictions. The objective of this work is to study the effects of temperature, expected under predictions of temperature increase on the development of the life cycle of the insect vector *R*. *prolixus* and on the metacyclogenesis of *T*. *cruzi* under controlled laboratory conditions.

## Materials and methods

### Study design

The *T*. *cruzi* scenarios of transmission have been previously studied by our group in Maní, Casanare (Colombia) and is characterized by dense forests of Palms. Infestation rates of 100% in a transect of 120 studied palms were reported [39]. Insect were collected manually in the the axils of *A*. *butyracea* palms and transported to our laboratory in the same day of capture.

Specimens of *R*. *prolixus* collected in *Attalea butyracea* palms were kept in incubators that simulated the same average annual humidity and temperature conditions of the axils of the palms where they were collected (26°C, 80% RH and photoperiod 12:12). Temperature was measured using an "EXTECH RHT 20: Humidity / Temperature Datalogger" in different palms. Previous field reports stated that the temperature in palms axils is 26°C-27°C according to Urbano et al. [40]. On the other hand, these devices were also used to measure and control the three temperatures (26, 28, 30°C) in the incubators, during the whole time of the study.

The triatomines maintained in the laboratory were fed chicken (*Gallus gallus*) blood once every 15 days. Veterinary medical services of Universidad de los Andes–Biological Sciences bioterium, (mice (*Mus musculus*) blood-chicken (*Gallus gallus*) blood).

### Temperature regimes

Taking into account the minimum temperature increase expected under climate change and the average temperature of the collection site, three temperatures were chosen for monitoring parasitemia in mice (*Mus musculus*) and metacyclogenesis in insect vectors: 26°C, 28°C and 30°C. These temperatures were adjusted in closed incubators provided with constant air flow, a constant relative humidity of 80% and a 12:12 light: dark photoperiod.

### Parasites

Taking into account that in Colombia, the most prevalent DTUs of *T*. *cruzi* are TcI and, less frequently, TcII, blood trypomastigotes of the strains MHOM/CO/04/MG (TcI) and MHOM/BR/53/Y (TcII) were used to conduct the experiments. These reference strains were previously characterized by 24 microsatellite markers and 10 mitochondrial markers (mMLST) and were maintained in successive passages from mouse to mouse every 15 days, following the recommendations of the Institutional Committee for the Care and Use of Laboratory Animals of the University of the Andes.

### Effects of temperature on the life cycle of *R*. *prolixus*

For each temperature, 4 groups of 30 newly oviposited eggs were formed. Each group was kept in a plastic container (Diameter 10.5 cm, Height 17 cm) with a filter paper base containing faeces of uninfected insects to ensure the presence of the microbiota in the insects to be studied. For each group, the mortality by stage, the average time to hatching, change to fifth instar and change to adult were recorded.

### Effects of temperature on fecundity and egg viability

For each temperature, 20 females and 20 virgin males obtained from the study of the life cycle were taken. The sample size was estimated for each experiment, taking into account the variability and the average of experimental data previously reported [41, 42]. The weights of the females were recorded before and after feeding as reported elsewhere [41], and reproductive pairs were formed, verifying copulation by observing the "spermatophore casing". This procedure was performed during 3 reproductive cycles, taking each cycle as the 21 days after feeding. The eggs oviposited per cycle were transferred to a Petri box with a filter paper base and were kept there to observe hatching. The *e-value* was calculated, as indicative of the capacity of a female to use the blood ingested towards egg production [43], as was the hatching rate for each pair in each reproductive cycle. The e-value was calculated as follows:

$$e-value=\frac{Total\;number\;of\;eggs\;laid\;21\;days\;post\;meal}{Initial\;female\;weight\;x\;blood\;meal\;weight}\;x\;1000$$ (1)

### Inoculation of ICR-CD-1 mice

Sixty mice were inoculated intraperitoneally with 0.2 mL of infected blood with each of the selected *T*. *cruzi* strains. Fifteen days post-inoculation, the mice were anaesthetized intraperitoneally with pentobarbital and were then exsanguinated by cardiac puncture. This procedure was previously approved by the research ethics committee and CICUAL of the University of the Andes in ruling 318 of 2014. The concentration of parasites in the blood and their viability were verified by light microscopy using a Neubauer chamber. If necessary, the infected blood was diluted in pathogen-free mouse blood to a concentration of 1×10 ^6^ parasites/ml.

### Effects of temperature on the metacyclogenesis of *T*. *cruzi* in *R*. *prolixus*

**Fig 1:**
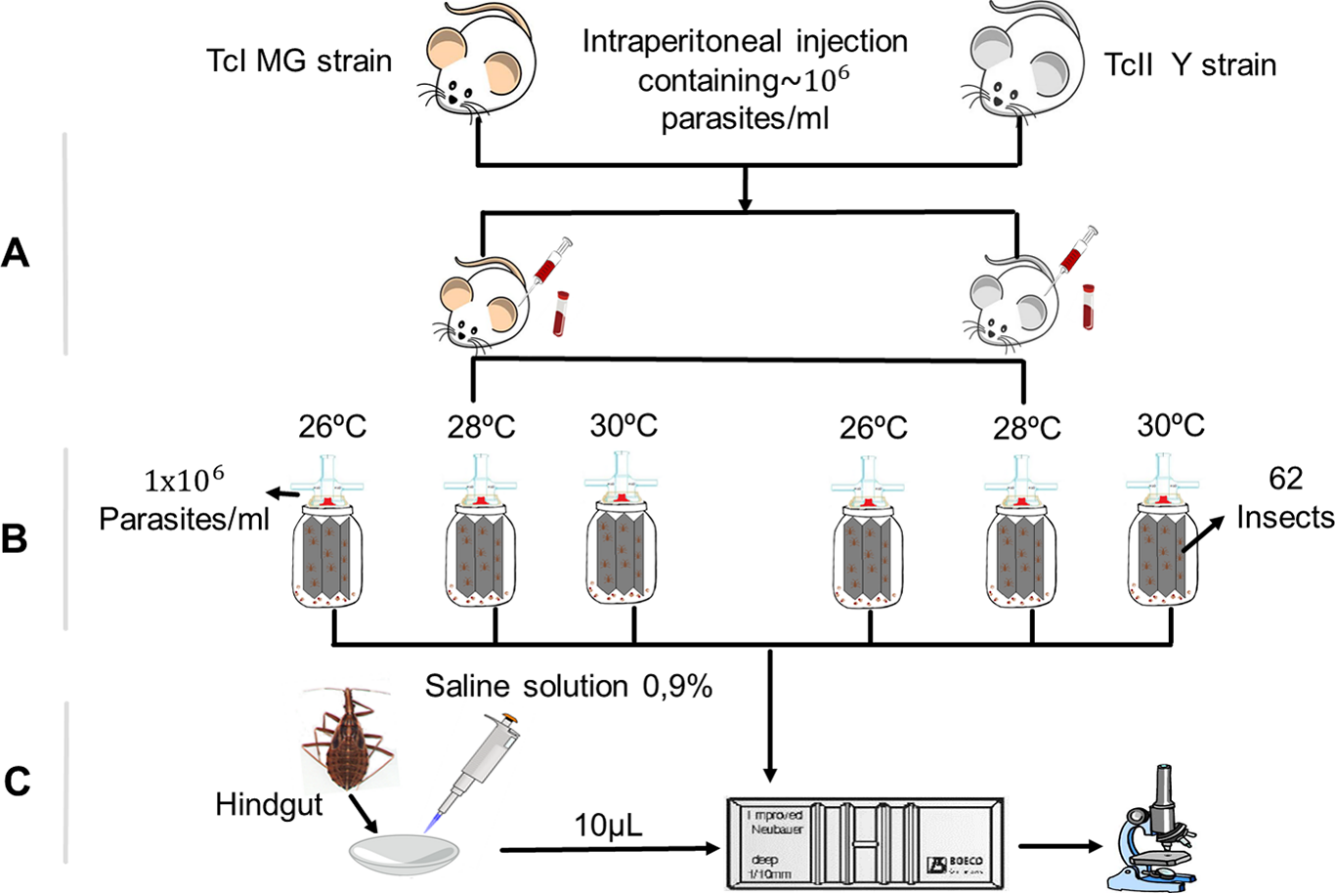
Methodology used to study the effect of temperature on the metacyclogenesis of T. cruzi in *R*. *prolixus*. (A) Inoculation of mice with strains of the parasite. (B) Artificial nymph feeding. (C) Analysis of trypomastigotes in the rectal ampulla and count in the Neubauer chamber.

For each of the selected strains, 186 fifth-instar nymphs of *R*. *prolixus* were fed heparinized mouse blood at a concentration of 1×10 ^6^ parasites/mL using an artificial feeder. After feeding, these nymphs were separated into groups of 62 individuals and were subjected to each of the temperatures. (Fig 1)

To establish the possible differences in the time at which metacyclic trypomastigotes were observed in the rectal ampulla, 2 nymphs were dissected per temperature and strain, from the second post-infection day and at intervals of 2 days until day 20. Similarly, to determine if there were differences between temperatures in the number of infectious forms in the rectal ampulla, 2 nymphs were dissected daily per temperature and strain from week 6 to week 8 post-infection. To perform the dissection, a cut was made in the last abdominal segment of the insect, and the rectal ampulla was carefully separated from the rest of the intestinal tract. The contents of the ampulla were macerated and resuspended in 100 mL of physiological saline (0.9%) at room temperature. Ten microlitres of this solution was used to quantify the number of metacyclic trypomastigotes in the Neubauer chamber.

### Ethics statement

All procedures with animals were conducted according to the Guide for the care and use of laboratory animals (8 ed)–National Research Council EEUU and the Institutional Animal Care and Use Committee Guidebook of OLAW. The Universidad de los Andes APLAC, in ruling 318 of 2014, approve all animals protocols used in the present work.

### Statistical analysis

All of the statistical analyses were performed in GraphPad Prism 7 software. First, the normality of the data was evaluated using the Shapiro-Wilk test; when the data were not normal, non-parametric tests were used. The Kruskal-Wallis test was used to evaluate the effects of temperature on the life cycle, fecundity and viability of *R*. *prolixus* eggs. Dunn’s multiple comparisons post hoc test was used to determine which temperatures were responsible for the significant difference found. In addition, a Chi-square test was perform to establish if there were differences in the mortality of the insects subjected to different temperatures. A Krustal-Wallis test was performed to evaluate if there were statistically significant differences between the temperatures evaluated and the DTUs used. Two tests were necessary, before 20 days post-infection (DPI) and after 35 DPI. Additionally, followed by an analysis of multiple comparisons to determine the groups that showed these differences.

## Results

### Effects of temperature on the life cycle of *R*. *prolixus*

**Fig 2:**
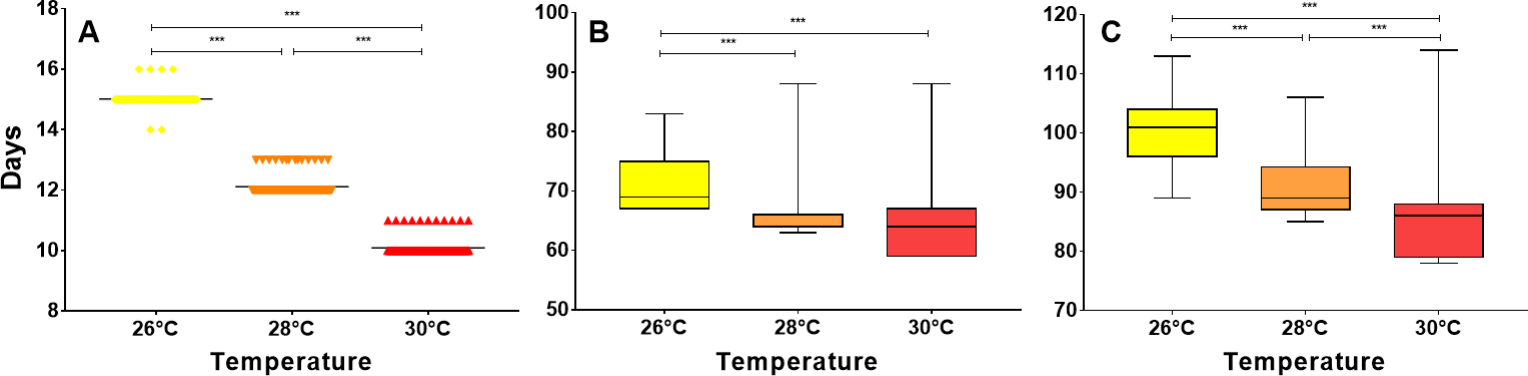
Effects of temperature on the life cycle of *R*. *prolixus*. (A) Hatching time by temperature. (B). Time needed to change to fifth instar nymph by temperature. (C) Time to change to adult by temperature. * = P < 0.05, ** = P < 0.01, *** = P < 0.001.

The time to hatching, change to fifth instar nymph and change to adult were significantly different between temperatures (Kruskal-Wallis test, P < 0.0001). In evaluating hatching time and time to adult, it was found that each temperature differed significantly from the other (Dunn's test, P < 0.0001). However, for the time required to change to fifth instar, significant differences were only found between 26°C and other temperatures (Dunn's test, P < 0.0001), but not between 28°C and 30°C. Compared with 26°C, the control temperature, the development time from egg to adult was reduced by 13% when the insects were kept at 30°C and by 9% when the eggs were kept at 28°C (Fig 2).

No significant differences were found in insect mortality between temperatures (Chi-square P = 0.67). In general, mortality was low for all temperatures (6.6% for 30°C, 5.83% for 28°C and 4.16% for 26°C) in comparison with previous studies [44], and was introduced especially during the change from fifth instar to adult, representing 75% of the mortality at 30°C, 83.3% at 28°C and 60% at 26°C.

### Effect of temperature on the fertility and fecundity of females

**Fig 3:**
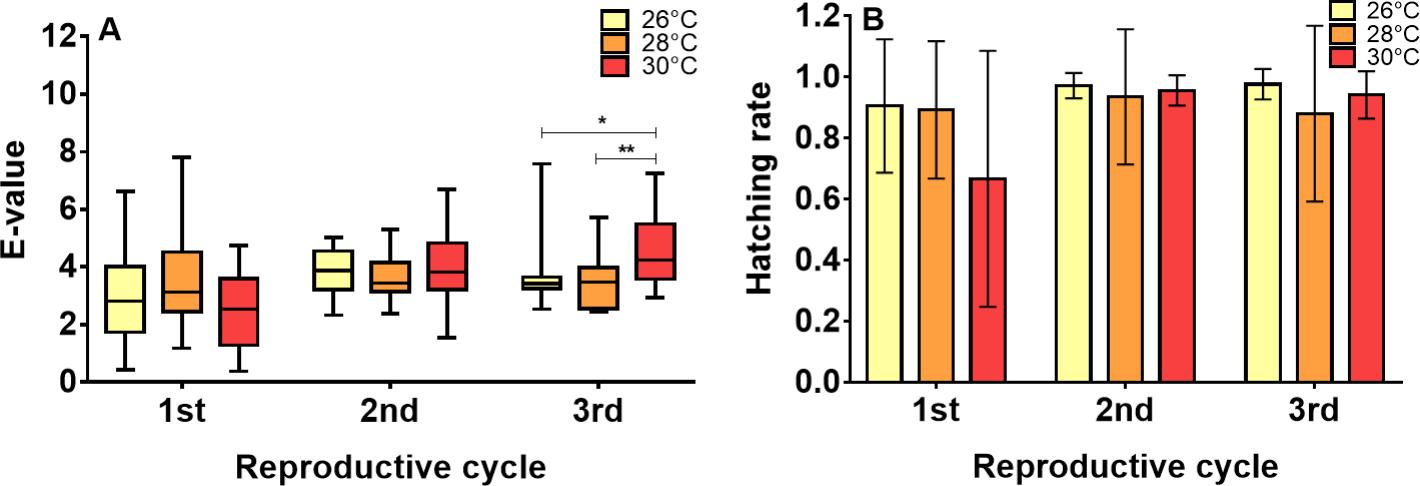
Effects of temperature on fecundity and *R*. *prolixus* egg viability. (A) Comparison of the e-values between temperatures in the first 3 reproductive cycles (B) Effect of temperature on the hatching rate in the first 3 reproductive cycles. Each reproductive cycle includes the 21 days after a feeding. * = P < 0.05, ** = P < 0.01, *** = P < 0.001.

The *e-value*, as an indicator of fecundity of the females, was not significantly different between reproductive cycles for the insects subjected to 26°C (Kruskal-Wallis test, P = 0.1080) and 28°C (Kruskal-Wallis test, P = 0.7409). At 30°C, the reproductive cycle did affect the *e-value* (Kruskal-Wallis test, P = 0.0001), with the first cycle being shorter and significantly different from the second (Dunn's multiple comparisons test, P = 0.0083) and third cycles (Dunn's multiple comparisons test, P = 0.0001). No significant differences were observed in the *e-values* between temperatures during the first (Kruskal-Wallis test, P = 0.2367) and second reproductive cycles (Kruskal-Wallis test, P = 0.3118), but there were differences during the third (Kruskal-Wallis test, P = 0.0043), with 30°C significantly different from 26°C (Dunn's multiple comparisons test, P = 0.0092) and 28°C (Dunn's multiple comparisons test, P = 0.0092) (Fig 3A).

However, the hatching rate was not affected by either the reproductive cycles (Kruskal-Wallis test, 26° P = 0.0533, 28° P = 0.1687 and 30°C P = 0.2032) or the temperatures to which the eggs were subjected (Kruskal-Wallis test, 26° P = 0.3689, 28° P = 0.2511 and 30°C P = 0.1333) (Fig 3B).

### Effects of temperature on the metacyclogenesis of *T*. *cruzi* in *R*. *prolixus*

**Fig 4:**
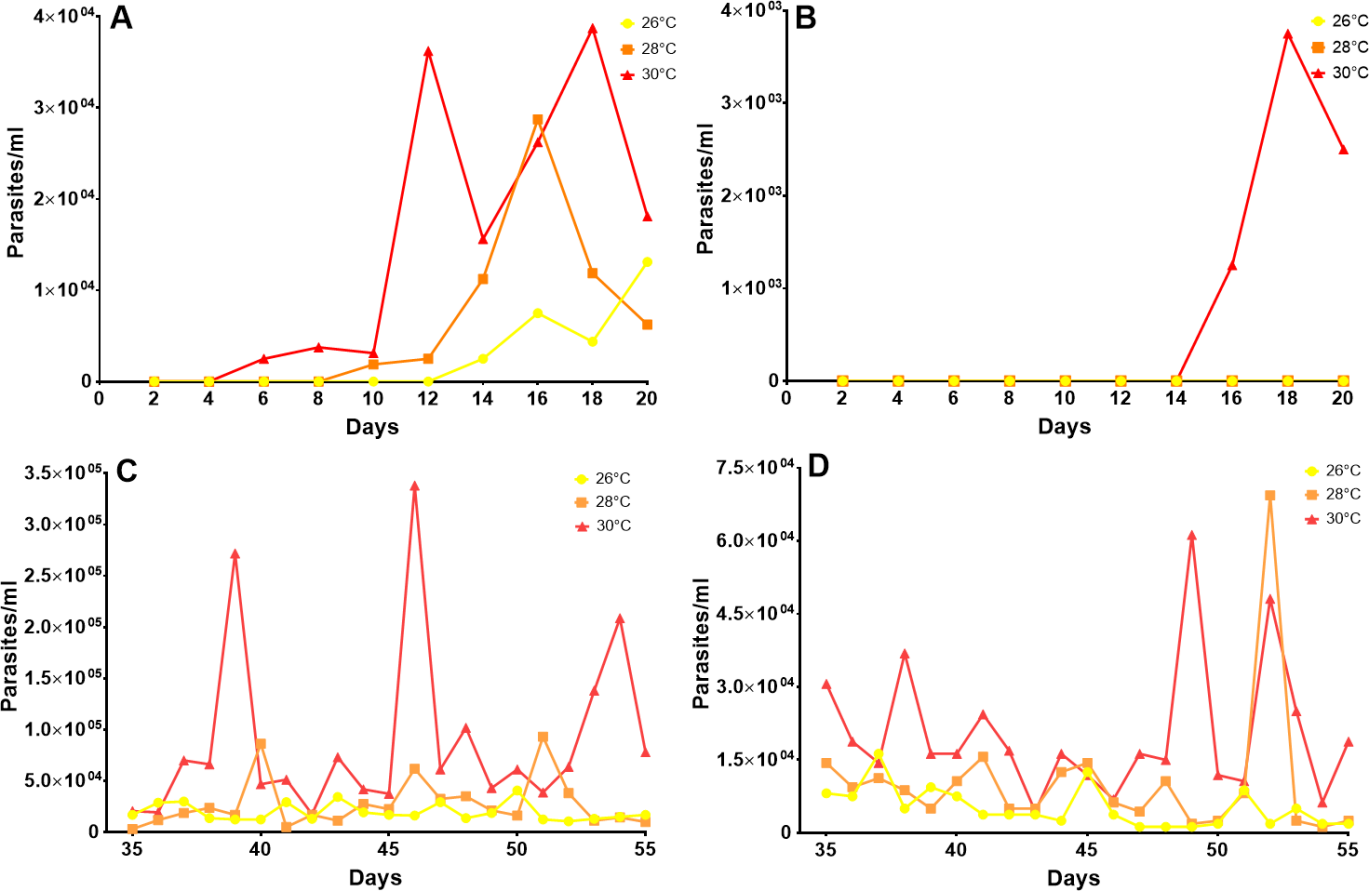
Effects of temperature on the metacyclogenesis of *T*. *cruzi*. (A) Amount of metacyclic tryomastigotes of TcI in the rectal ampulla of *R*. *prolixus* by temperature, 20 days post-infection. (B) Amount of metacyclic tryomastigotes of TcII in the rectal ampulla of *R*. *prolixus* by temperature, 20 days post-infection. (C) Amount of metacyclic tryomastigotes of TcI in the rectal ampulla of *R*. *prolixus* from week 6 to week 8 post-infection by temperature. (D) Amount of metacyclic tryomastigotes of TcII in the rectal ampulla of *R*. *prolixus* from week 6 to week 8 post-infection by temperature.

For TcI, the appearance of metacyclic trypomastigotes was observed in the rectal ampulla on the sixth day post-infection at 30°C, while at 26°C and 28°C, it was observed on days 10 and 14, respectively. (Fig 4A). Similarly, for TcII, the appearance of infective forms was observed on day 16 post-infection at 30°C, while at 28°C and at 26°C, metacyclic trypomastigotes were not observed during the first 20 days post-infection. (Fig 4B). All the data evaluated here exhibited a non-parametric distribution. Then, a Krustal-Wallis test was used to evaluate the difference between temperatures and DTUs. A first analysis carried out up to 20 DPI showed that there was no statistically significant difference in the concentration of parasites between the temperatures analyzed for each DTUs, and the same when making a comparison between both DTUs. Otherwise it happened with the analysis carried out after the 35 DPI, where a statistically significant difference of the concentration of parasites was observed between the temperatures analyzed for both DTUs (p = <0.0001). When performing the multiple comparisons test, the temperature of 30° C presented a clear difference with respect to 26° and 28°, given by an increase in the concentration of parasites. These results were maintained for both DTUs. An analysis between DTUs showed a higher concentration of parasites in the insects infected with the TcI DTU with respect to TcII. These results were verified statistically (p = <0.0001), and were maintained throughout the three temperatures.

From weeks 6 to 8 after inoculation, there were significant increases in the numbers of infective forms of TcI and TcII found in the rectal ampulla of insects subjected to 30°C compared with insects subjected to 26°C and 28°C (Fig 4C and 4D). In general, a much greater amount of metacyclic trypomastigotes of the TcI strain compared with the TcII strain was observed. (Fig 4)

## Discussion

In this study, the effects of increasing temperature, as expected under predictions of climate change, on the life cycle, fecundity and viability of *R*. *prolixus* eggs and on the development of *T*. *cruzi* in *R*. *prolixus* were evaluated. Our results indicate that increasing temperature from 26°C to 30°C has effects on the time of development (Fig 2), the fecundity of the insect (Fig 3A) and the development of the parasite (Fig 4). However, no significant effect on egg viability was observed (Fig 3B).

The negative relationship between the temperature and the development time of *R*. *prolixus* obtained was in agreement with findings previously reported by Clark [45] and Luz et al [46]. This faster development of insects can be a result of increased metabolic rate caused by the increase in temperature [47], a relationship already demonstrated for *R*. *prolixus* [48]. In Fig 2B and 2C, showing the results for changes to fifth instar and to adult, one can see that the times to such changes in some insects are equal to those for other temperatures. These observations could be explained by the frequency of feeding and the amount of blood ingested by each specific individual. Although a food source was offered weekly, it was observed that some insects did not feed or did not ingest enough blood to change, which generates an increase in the life cycle duration [18].

The temperature at which the greatest mortality was observed was 30°C; however, the percentage was similar to that reported by Arevalo et al [41] and was less than observed by Gomes et al [49] for *R*. *prolixus* under laboratory conditions. In general, the greatest mortality occurred at the change to fifth instar, which has been reported by other investigators for *R*. *prolixus* [41, 49,46] and other triatomine species, such as *R*. *robustus* [50], *T*. *infestans* [51] and *Meccus picturatus* [52]. Despite of our results, acclimation capability to temperature must be considered. This is a long-step process that might take several years and in our study we were not able to consider that variable. This is relevant in the light of previous studies that show the three main sensitive parameters for Chagas disease transmission (mortality rate, density of vectors and bite rate) [53]. We explored two of them (mortality and density). However, in future studies the bite rate must be considered because with the increase of temperature, there will occur a higher metabolic rate and in consequence a higher bite rate which could drive the increase in the transmission of *T*. *cruzi*. Therefore, in the future bite rate should studied.

Although the presence of Spermatophore casings was checked to verify copulation, the number of copulations per couple was not taken into account. Therefore, it is possible that both the differences observed in the *e-value* between reproductive cycles for 30°C and the low hatching rate observed during the first reproductive cycle at this temperature can be explained by a difference in the number of copulations. For *T*. *brasiliensis*, it is known that females that have multiple copulations produce more eggs with a greater percentage of fertility than females that copulate only once [54]. However, it would be interesting to study fluctuations in fecundity during more reproductive cycles to establish if the differences observed in the third reproductive cycle are maintained or if they are only the result of the number of copulations. If these differences were maintained between reproductive cycles, it would be possible to think that at 30°C, the number of eggs laid per female would increase, and as a consequence, there would be a greater number of insects per reproductive cycle that would be available to transmit *T*. *cruzi*. This has been studied by Schilman and Lazzari in 2004 [55], where they found that females oviposit across a range of temperatures from 22 to 33°C with a peak at 25–26°C in accordance with our findings. Nevertheless, they cannot discern whether *R*. *prolixus* females actively choose certain oviposition substrates according to temperature or whether they oviposit where they find themselves. These results, both in the life cycle and in the fecundity and fertility of the insects, suggest that the expected temperature increase under climate change could increase the insect density in the palms of *A*. *butyacea*. This possibility is not very different from field reports that state that the density of insects in these palms is higher in summer seasons than in rainy seasons, when the temperature decreases [39].

The time at which infectious forms of both TcI and TcII are observed in the rectal ampulla of *R*. *prolixus* is shorter at 30°C than at the other temperatures. These results are consistent with previous data reported for *Triatoma infestans*, where metacyclic trypomastigotes were observed to be faster at 28°C than at 20°C [56]. Likewise, the number of infective forms observed for TcI from week 6 to week 8 was greater at 30°C than at the other temperatures. This relationship between the temperature and reproduction of the parasite has been previously reported under *in vitro* conditions for the epimastigote stage [43,57]; however, it must be taken into account that under *in vitro* conditions, the parasite is not subject to the immune factors and the microbiota of the insect [58]; therefore, it is difficult to extrapolate and compare the *in vitro* results with the results obtained in this study.

The amount of metacyclic trypomastigotes observed in the rectal ampulla of *R*. *prolixus* was markedly higher for TcI than for TcII. This difference is likely due to the presence of trypanolytic factors in the haemolymph of *R*. *prolixus* that differentially affect TcII but not TcI, which is the main reason why this species of vector is considered to lack the capacity to transmit said DTU [59, 60]. However, it is interesting to note that the temperature may be affecting this interaction, since at 30°C, an increase in the number of infective forms was observed for TcII (Fig 4B–4D). Therefore, in the future, it would be important to evaluate whether this increase in the number of infective forms, due to temperature, could enhance the capacity of *R*. *prolixus* to transmit *T*. *cruzi* II.

In conclusion, this study showed that as temperature increases (26 to 30°C), there is a more rapid appearance and an increase in the number of infective forms of *T*. *cruzi* in *R*. *prolixus*, along with a significant decrease in the development time of said vector. These results could suggest that under the effects of climate change, the probability of infection with *T*. *cruzi* could increase. However, it is necessary to study the effects of more climatic and ecological factors and the effects of such factors on parasite-vector interactions to predict the future of Chagas disease with better accuracy.

## Supporting information

S1 Database Includes the life cycle, hatching rate, fertility and parasite counting for each temperature. (XLSX)
